# Optimizing disordered crystal structures

**DOI:** 10.1107/S2053229623001547

**Published:** 2023-02-21

**Authors:** Anthony Linden

**Affiliations:** a Department of Chemistry, University of Zurich, Winterthurerstrasse 190, CH-8057 Zurich, Switzerland

**Keywords:** disorder, disorder modelling, whole molecule disorder, commentary

## Abstract

A mixed-crystal full-mol­ecule disorder situation is revisited in Parkin *et al.* [*Acta Cryst.* (2023), C**79**, 77–82]. A new inter­pretation of the data leads to the conclusion that the crystal structure is more likely to be a three-component superposition of enantiomers and the *meso* isomer of an organic compound and the article is thus a good learning example for dealing with a highly disordered structure.

Disordered crystal structures constitute about 28% of the entries in the Cambridge Structural Database (Groom *et al.*, 2016 ▸). It is always exciting when a new data set comes off the diffractometer and one hopes for a rapid refinement of what is expected to be a routine structure determination, but then the discovery of disorder ruins one’s day when one realises that considerable time and effort will be needed to arrive at an optimal structure model. Sometimes the disorder is only present in a solvent mol­ecule or counter-ion, *e.g*. a tumbling PF_6_^−^ anion, where it is not so critical to develop a ‘perfect’ model, but nonetheless, every improvement in the disorder model for such species usually improves the agreement factors and the precision of the atomic coordinates for all components in the structure, thereby leading to more precise geometric parameters, even in the non-disordered main component. For many analyses, the geometric parameters are the most tangible results of inter­est, so obtaining their best precision is important.

In crystal structures where the mol­ecule or fragment of inter­est is disordered, the disorder might have no consequences for the understanding of the chemical species present, such as conformational variations in the puckering of a five-membered ring or in the orientation of a terminal –CF_3_ group. In such cases, modelling the disorder is often, but not always, relatively straightforward.

At the other extreme, the challenge of dealing with extensive, subtle or even full-mol­ecule disorder often requires much effort, because the positions of many atoms from the various disorder components are almost overlapping and it can be tricky to set up the best combination of restraints and/or constraints to allow the refinement to work smoothly while maintaining the correct chemical and geometrical logic of the species present. Full-mol­ecule disorder can occur when the topology and polarity of the compound is such that the mol­ecule can slot into its space in the crystal structure without caring about which way around it sits. A similar scenario can occur when more than one chemical species with similar topology is present in the crystal and they occupy the same crystallographic site; a mixture of diastereoisomers is one such example. Of course, these topological considerations are not necessarily limited to (entire) mol­ecular species. Whether it is a mol­ecular or an extended structure, a part of the asymmetric unit, such as a single ligand, might also fit into its cavity in more than one orientation or conformation.

A mixed-crystal full-mol­ecule disorder situation is described in this issue of *Acta Crystallographica Section C* by Parkin *et al.* (2023 ▸). The authors revisit the product of an organic reaction published by some of the same authors (Mohamed *et al.*, 2016 ▸), which at the time was described as the *meso* isomer of (*E*,*E*)-1,10-[1,2-bis­(4-chloro­phen­yl)ethane-1,2-di­yl]bis­(phenyl­diazene) disordered across a crystallographic centre of inversion. Some unusual geometry subsequently noticed in the original model prompted the re-evaluation. The new inter­pretation of the data leads to the conclusion that the crystal structure is more likely to be a three-component superposition of the *meso* form with, additionally, the *S*,*S* and *R*,*R* enanti­omers. Such a superposition is feasible given the spatial similarities of the isomers. The matter is made more complicated by the question of whether or not the true space group is *Cc* or the centrosymmetric *C*2/*c*.

While the motivation of Parkin *et al.* (2023 ▸) is to improve on the original inter­pretation of the structure, the means of getting there is presented in a clear and instructive way. The article is thus a good learning example for dealing with a highly disordered structure and for what some people call ‘forensic crystallography’. Not only do the authors contend with modelling the disorder, but also deduce which chemical compounds are most likely to be present and address the space-group ambiguities. Usually, this sort of problem requires many trial refinements and, in general, practitioners should not feel reluctant to try out various ideas before settling on their final model. The problem is approached from both crystallographic and chem­istry points of view. That is, the models for each of the disordered components have to be geometrically logical, as it is unlikely that such organic mol­ecules would be geometrically distorted. The models should also account for the observed electron-density distribution; the presence of Cl atoms in these mol­ecules makes their disordered positions stand out more clearly, thereby giving a starting point for developing the models, which understandably require a significant array of restraints and constraints. As with many complex disorder problems, there is probably not a unique way to select restraints and constraints to arrive at an acceptable model. The authors themselves state: ‘*it is unlikely that any two crystallographers would settle on the same combination of constraints/restraints*’. Finally, the chemical origin of the enanti­omers and *meso* isomer has to be understood – originally only the presence of the *meso* compound had been assumed. This highlights the importance of critical and logical thinking and a good dialogue between the crystallographer and the scientist(s) producing the substances and inter­preting the other spectroscopic data, if they are not the same person. These comments are not intended as a criticism of the original work – sometimes another pair of eyes and a different viewpoint reveal things that the others have not noticed.

Coincidentally, this issue of the journal reports another disordered mixed-crystal structure where the disorder is a consequence of the superposition of two disasteroisomers, one of which is additionally conformationally disordered, at the same crystallographic site in the crystal, treatment of which requires a three-component disorder model, although full-mol­ecule disorder is probably not present (Linden *et al.*, 2023 ▸).

Anecdotally, I was recently contacted by a young crystallographer who was struggling with the inter­pretation of a disordered structure that looked somewhat like the expected synthesis product, but was strange in other respects, especially some excessively long bonds. After discussing the synthesis of the material, we concluded that the crystal was a mixed-crystal composed of unreacted starting material and product, and the two species had crystallized randomly at the same crystallographic site. It was then possible to develop an appropriate disorder model for the structure, albeit still with some non-ideal geometry. I suggested that if it was at all possible still, some additional attempts at carefully optimizing the reaction and/or work-up and separation procedures might allow one to obtain the product in a more pure form. Indeed, the desired pure product was obtained subsequently and a clean crystal structure ensued, thus obviating the need to continue with or attempt to publish the mixed-crystal structure. Of course, the separation or purification of mixtures can sometimes be extremely difficult, but if tweaks to the procedures have not yet been tested, further attempts might be more fruitful than trying to continue with a messy, inferior quality, or even ambiguous, mixed-crystal structure determination.

Small-mol­ecule crystallographers are fortunate today to have some excellent tools available to assist with the modelling of disorder. While the program *SHELXL* (Sheldrick, 2015 ▸) has long had an extensive array of restraint and constraint instructions (SADI, SAME, DFIX, DANG, SIMU, DELU and RIGU, to name just a few), manually developing the initial disorder model was sometimes cumbersome. Nowadays, the *Olex^2^* software (Dolomanov *et al.*, 2009 ▸), as one example, has excellent and clever tools in the Graphical User Inter­face (GUI), which allow one, after a little practice, to split, drag and rotate fragments conveniently in order to obtain a good starting approximation to the disorder and then apply the appropriate restraints and/or constraints. The GUI of *ShelXle* (Hübschle *et al.*, 2011 ▸) can be used in a similar way. *Olex^2^* also has a fragment database, which can be used to help model common fragments or solvent mol­ecules. Excellent YouTube tutorials demonstrating many of the features of both of these programs, not just disorder handling, are available online.

Further discourses on many aspects of obtaining the best results from a crystal structure determination, including the handling disordered structures, can be found in Vinaya *et al.* (2023 ▸), Clegg (2019 ▸) and Linden (2020 ▸).
